# *Cryptococcus neoformans* Δ*sgl1* Vaccination Requires Either CD4^+^ or CD8^+^ T Cells for Complete Host Protection

**DOI:** 10.3389/fcimb.2021.739027

**Published:** 2021-09-08

**Authors:** Tyler G. Normile, Antonella Rella, Maurizio Del Poeta

**Affiliations:** ^1^Department of Microbiology and Immunology, Stony Brook University, Stony Brook, NY, United States; ^2^Division of Infectious Diseases, School of Medicine, Stony Brook University, Stony Brook, NY, United States; ^3^Veterans Administration Medical Center, Northport, NY, United States

**Keywords:** *Cryptococcus neoformans*, cryptococcosis, host immune response, immunodeficiency, vaccination, sterylglucosides, immunoadjuvant, T cells

## Abstract

*Cryptococcus neoformans* is a fungal pathogen causing life-threatening meningoencephalitis in susceptible individuals. Fungal vaccine development has been hampered by the fact that cryptococcosis occurs during immunodeficiency. We previously reported that a *C. neoformans* mutant (Δ*sgl1*) accumulating sterylglucosides (SGs) is avirulent and provides complete protection to WT challenge, even under CD4^+^ T cell depletion, an immunodeficient condition commonly associated with cryptococcosis. We found high levels of SGs in the lungs post-immunization with Δ*sgl1* that decreased upon fungal clearance. Th1 cytokines increased whereas Th2 cytokines concurrently decreased, coinciding with a large recruitment of leukocytes to the lungs. Depletion of B or CD8^+^ T cells did not affect either Δ*sgl1* clearance or protection from WT challenge. Although CD4^+^ T cell depletion affected clearance, mice were still protected indicating that clearance of the mutant was not necessary for host protection. Protection was lost only when both CD4^+^ and CD8^+^ T cells were depleted, highlighting a previously unexplored role of fungal-derived SGs as an immunoadjuvant for host protection against cryptococcosis.

## Introduction

Given the rise in lymphopenia due to HIV/AIDS, solid organ transplant, chemotherapy (Li et al., 2019; Fierer, 2019; Pathakumari et al., 2020), and immunosuppressive therapies to control chronic diseases (Grebenciucova et al., 2016; Ward et al., 2016; Bryan et al., 2020), opportunistic invasive fungal infections have emerged as a global health concern, killing ~1.5 million people per year. Among these opportunistic fungal infections, cryptococcosis is caused by *Cryptococcus neoformans* and *Cryptococcus gattii* (Chang and Chen, 2015; Diaz, 2020; Montoya et al., 2021), resulting in ~220,000 new cases and ~180,000 deaths annually (Rajasingham et al., 2017; Mayer and Kronstad, 2020).

Once the environmentally endemic *Cryptococcus* spores are inhaled into the lungs, the initial infection is rapidly controlled within lung granulomas in immunocompetent hosts (Shibuya et al., 2005; Brunet et al., 2018). During immunodeficiency, however, the fungus can disseminate to the brain to cause lethal meningoencephalitis. The mortality rate can be attributed to the aggressiveness of the fungus as well as limited antifungal treatment options available to combat cryptococcosis, resulting in high rates of antifungal resistance [reviewed in (McEvoy et al., 2020)] and host toxicity (Bicanic et al., 2015). Despite extensive vaccine development research, no fungal vaccines are currently available for clinical use.

The fungal vaccine development pipeline has explored various antigens, adjuvants, vaccination routes and frequencies, as well as vaccine-inducing mutants to control cryptococcosis, yielding differing levels of host protection [reviewed in (Scorzoni et al., 2017; Caballero Van Dyke and Wormley, 2018; Nami et al., 2019; Ueno et al., 2020; Pathakumari et al., 2020)]. As current fungal vaccine development is not rooted in models of immunodeficiency, there is a major bottleneck in advancing preclinical findings to prevent these opportunistic infections in the clinical setting. To identify the host cell subtypes that may confer protection against opportunistic invasive fungal infections, the efficacy of fungal vaccines should be tested under immunodeficiencies specific to these infections. Cryptococcosis occurs under CD4^+^ T cell immunodeficiency and our previous work showed that immunization of a CD4^+^ deficient host with *C. neoformans* Δ*sgl1* mutant accumulating sterylglucosides (SGs) conferred total protection against a lethal challenge with the highly virulent *C. neoformans* wild-type (WT) H99 strain (Rella et al., 2015).

SGs are known immunomodulatory glycolipids that have been used in a wide variety of therapeutic applications [reviewed in (Grille et al., 2010; Normile et al., 2020b)]. They have been shown to be promising immunoadjuvants for protection against pulmonary tuberculosis in human clinical trials (Donald et al., 1994), enhancement of human T cell differentiation *in vivo* (Bouic et al., 1996), and protection against mouse models of invasive candidiasis (Lee and Han, 2006; Lee et al., 2007). Collectively, these studies suggest that SGs induce T cell proliferation, direct Th1 cell differentiation, and induce earlier host recognition leading to rapid recruitment of innate immunity. However, the majority of the vaccination studies utilizing SGs have been performed using plants (Thuan et al., 2013; Singh et al., 2016; Castillo et al., 2019) or purified SGs as a standalone compound prior to infection to induce a protective immune response, not as a simultaneous immunoadjuvant present during the active infection stage (Lee and Han, 2006; Lee et al., 2007).

In the present study, we provide a comprehensive analysis of the host immune response against the live, attenuated *C. neoformans* Δ*sgl1* mutant during both the immunization phase and the challenge phase under various host immunodeficiencies. We found that our SG-accumulating mutant induced a robust pro-inflammatory lung environment with increased leukocyte recruitment to the lungs. During immunosuppressive conditions, the host was able to eliminate *C. neoformans* Δ*sgl1* from the lungs when monocytes, macrophages, and/or neutrophils as well as B or CD8^+^ T cells were depleted. Even when macrophages, B cells, or CD8^+^ T cells depleted, mice were still fully protected against a subsequent WT infection. Although mice depleted of CD4^+^ T cells were unable to eliminate *C. neoformans* Δ*sgl1* from the lungs, they showed no signs of morbidity or fungal dissemination and were fully protected against WT infection during which time they clear the mutant strain. Only when both CD4^+^ or CD8^+^ T cells were depleted, mice were unable to clear *C. neoformans* Δ*sgl1* and lost complete protection against the WT infection. Our findings emphasize that *C. neoformans* Δ*sgl1* induces robust host immunity and highlight a previously unexplored role of SGs as potent immunomodulators for protection against cryptococcosis during severe immunodeficiency.

## Materials and Methods

### Fungal Strains

The fungal strains used in this study were *C. neoformans* var. *grubii* strain H99 serotype A (WT) and *C. neoformans* Δ*sgl1*, a mutant strain accumulating SGs developed by our group (Rella et al., 2015). Fungal strains were freshly recovered from a -80°C freezer stock on yeast extract, peptone, and dextrose (YPD) plates at 30°C. An isolated colony was added to 10ml of YPD broth and grown for 16-18h at 30°C with shaking, washed thrice in sterile PBS, counted, and resuspended in sterile PBS at the desired concentration.

### SG Quantification and Purification of EVs

*C. neoformans* WT strain H99 or Δ*sgl1* mutant were grown in yeast nitrogen base (YNB) for 24h at 30°C with shaking and lipid extraction was performed on pelleted 5x10^8^ cells. The extraction followed three major steps: Mandala’s method (Mandala et al., 1995), followed by the methodology of Bligh and Dyer (Bligh and Dyer, 1959), and then base hydrolysis (Singh et al., 2011). The samples obtained were resuspended in chloroform:methanol (2:1) for the thin layer chromatography (TLC) and compared to a standard of known SGs as reference. For mass spec (MS) analysis, ergosteryl-3-β-glucosides (SGs) were analyzed as previously described (Munshi et al., 2018). The samples were resuspended in 1mM of ammonium formate and 0.2% formic acid–methanol. The separation was processed through a Thermo Accela high-performance liquid chromatography (HPLC) system (San Jose, CA). The HPLC system was attached to the heated electrospray ionization (HESI) source of a Thermo TSQ Quantum Ultra triple quadrupole mass spectrometer (San Jose, CA). Data were analyzed on Thermo Xcalibur 2.2 Quan Browser software and normalized to inorganic phosphate content (Munshi et al., 2018).

Extracellular vesicle (EV) isolation from *C. neoformans* WT strain H99 or Δ*sgl1* mutant was performed following the protocol of Rodrigues et al. (Rodrigues et al., 2016). Briefly, the cultures were grown in YNB for 48h and subsequently centrifuged at 4k, 10k, and 15k*xg* for 15min each to remove cellular debris. After the final spin, the supernatant was concentrated using an AMICON Pro Purification System with 100kDa Ultra-0.5 Device (Millipore Sigma). The concentrated supernatant was then ultracentrifuged at 100k*xg* to isolate EVs. Sterol quantification within the EVs was performed using an Amplex Red kit (Thermo Fisher Scientific) and SG quantification was performed using liquid-chromatography MS (LC-MS).

### Mice and Ethical Statement

Male and female CBA/J mice were purchased from Envigo. Female TCR β KO mice and C57BL/6 were purchased from Jackson Laboratory. All animals had access to food and water *ad libitum*. All animal procedures were approved by the Stony Brook University Institutional Animal Care and Use Committee (protocol no. 341588) and followed the guidelines of the American Veterinary Medical Association.

### Infection, Survival Studies, Fungal Burden, and *In Vivo* Depletions

Primary infections were carried out in mice 4-6 weeks of age, and WT infections in mice 7-9 weeks of age. For all infections, mice were anesthetized with xylazine/ketamine solution intraperitoneally (IP) (95mg of ketamine and 5mg of xylazine per kg of animal body weight) and infected intranasally (IN) with 5x10^5^ yeast in 20μl of PBS. For survival analysis, animals were immunized with *C. neoformans* Δ*sgl1* or mocked-treated with sterile PBS, challenged 30 days later with WT *C. neoformans* H99, and monitored daily until the experimental endpoint. Animals that appeared moribund or had lost more than 20% body weight were euthanized using CO_2_ inhalation. For fungal organ burden analysis, mice were euthanized via CO_2_ inhalation on specified timepoints, the lungs, brain, spleen, kidneys, and liver were removed, homogenized in 10ml of sterile PBS using a Stomacher 80 blender (Seward, UK), and dilutions were grown on YPD plates at 30°C for 72h. For *in vivo* cell depletion, all antibodies were purchased from BioXCell, and clodronate-loaded liposomes were purchased from Encapsula Nano Sciences. Depletions were performed 48h prior to infection and continued the length of the experiment to maintain depletion. The specifics of each reagent can be found in **Table 1**. All antibodies were isotype-matched for control groups or empty lysosomes (vehicle) used for clodronate-loaded liposomes.

**Table 1:** Reagents used for *in vivo* depletions.

Antibody/chemical	Clone	Concentration	Administration	Targeted population
Anti-Ly6G	1A8	225μg/100μl	Every 48h IP	Neutrophils
Anti-Gr1	RB6-8C5	250μg/100μl	Every 48h IP	Monocytes/neutrophils
Anti-CD19	1D3	300μg/100μl	Every 120h IP	B cells
Anti-CD8	116-13.1	400μg/100μl	Every 120h IP	CD8^+^ T cells
Anti-CD4	GK1.5	400μg/100μl	Every 120h IP	CD4^+^ T cells
Clodronate	–	250μg/50μl	Every 72h IN	Alveolar macrophages

IP, intraperitoneally; IN, intranasal.

All depletion treatment administrations began 48 hours prior to immunization or infection.

### Flow Cytometry and Cytokine Quantification

For quantification of immune populations, lungs were aseptically excised from euthanized mice, minced into fluorescence activated cell sorter (FACS) buffer supplemented with collagenase IV and DNase I, homogenized through a 70μm pore filter, red blood cells lysed in Ack lysis buffer, washed with PBS, and filtered into single cell suspensions in FACS buffer. Cells were counted and 10^6^ cells were Fc blocked for 20 mins at 4°C in the dark and surface stained using various antibody cocktails of direct fluorochrome-conjugated antibodies for 30 mins at 4°C in the dark. The antibody-fluorochrome combinations used were as follows: viability-AF700; CD45-BV711; CD4-BV785; CD8-BV650; F4/80-APC; Ly6C-APC-Cy7; CD11b-BV510; Ly6G-PE; SiglecF-PE TexasRed; CD11c-PE-Cy7; CD24-PerCP-Cy5.5; MHC II-FITC; CD19-BV421. The detection of cytokines in the lung tissue was analyzed using a Milliplex Mouse Cytokine/Chemokine Magnetic Bead Premixed 25 Plex kit (Millipore Sigma), following the 2h incubation protocol by the manufacturer. The samples were immediately run on a Bioplex 200 system running Bioplex Manager version 4.1.1.

### Histology

For histological analysis, mice were euthanized at indicated time points. Lungs were removed, washed with PBS, and fixed in 10% formalin solution for 24 hours. The lungs were then dehydrated in ethanol, infiltrated with paraffin, sliced, and stained with hematoxylin and eosin (H&E) or mucicarmine as indicated in the figure legends. Three lungs were analyzed for each group at each timepoint, and a representative image for each group is shown.

### Statistical Analysis

Statistical analysis was described in each figure caption. The Mantel-Cox log-rank test was used to calculate significance for survival studies. Two-tailed unpaired *t* tests were used to calculate statistical significance between two samples, and two-way ANOVA using Šídák’s multiple comparisons test for *P*-value adjustment was used to calculate statistical significance between more than two samples using GraphPad Prism 9 software. Statistical significance was defined in this study as *P < 0.05; **P < 0.01; ***P < 0.005; ****P < 0.001.

## Results

### Sterylglucosides Are Found Extracellularly Both *In Vitro* and *In Vivo*

**Figure 1:**
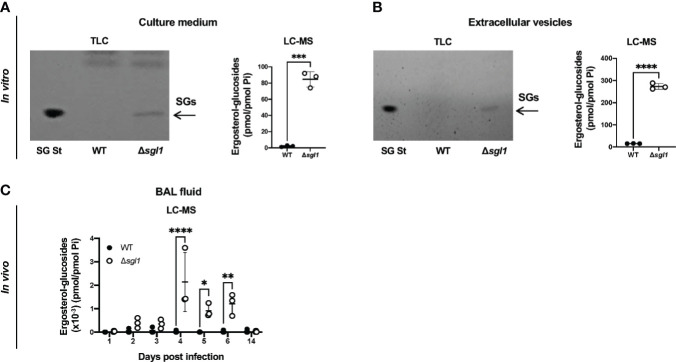
Ergosterol-glucosides (SGs) can be detected both *in vitro* and *in vivo* with *C neoformans* (*Cn*) *Δsgl1* but not the WT strain. **(A, B)** *In vitro* analysis of the growth medium **(A)** or extracellular vesicles **(B)** for SGs from cultures of either *Cn* WT or *Δsgl1* was performed by TLC and LC­-MS. **(C)** *In vivo* analysis of the bronchoalveolar (BAL) fluid of CBA/J mice infected with either Cn WT or Δ*sg*/*1* for SGs was performed by LC-MS over the indicated timeline (n=3 mice/strain/timepoint). Significance was determined by a two-tailed unpaired t-test **(A, B)** or a two-way ANOVA using Šídák’s multiple comparisons test for *P* value adjustment **(C)**. Graphed data represents the mean +/- SD, and significance is denoted as *P < 0.05; **P < 0.01; ***P < 0.005; ****P < 0.001.

Our previous work uncovered that deletion of the sterylglucosidase gene causes an accumulation of SGs in *C. neoformans* Δ*sgl1* cells (Rella et al., 2015). We first asked whether these SGs were solely present inside the yeast cells or also secreted extracellularly. We found that *C. neoformans* Δ*sgl1* secreted SGs into the culture medium, both as free molecules (**Figure 1A**) and associated with fungal extracellular vesicles (**Figure 1B**). In contrast, we did not observe any detectable SGs in *C. neoformans* WT culture medium or vesicles. We then evaluated the presence of SGs in mice and found SGs in the bronchoalveolar lavage (BAL) fluid after IN inoculation of *C. neoformans* Δ*sgl1* but not the WT strain (**Figure 1C**). SGs were detected as early as 2 days post inoculation with *C. neoformans* Δ*sgl1* and significantly greater accumulation on days 4, 5, and 6 compared to the WT strain. SGs returned to undetectable levels by day 14 post inoculation (**Figure 1C**) suggesting that SGs were only associated with the presence of *C. neoformans* Δ*sgl1* since the host clears the mutant by day 14 post inoculation. These data suggest that SGs can be found extracellularly *in vitro* and *in vivo*, providing an opportunity to impact the host immune response.

### *C. neoformans Δsgl1* Drives a Pro-Inflammatory Lung Cytokine Profile in the Host

**Figure 2:**
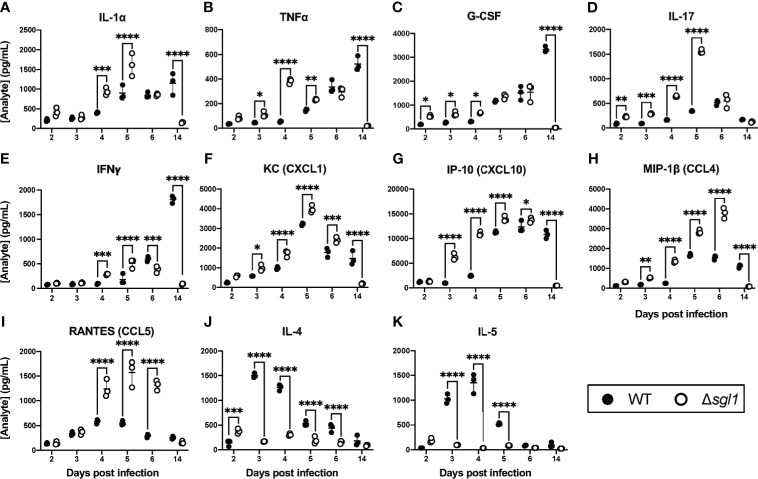
*C. neoformans (Cn)* Δ*sgl1* induces a pro-Inflammatory lung cytokine microenvironment in the host. CBA/J mice were intranasally infected with 5x10^5^ CFU of either WT or *Cn* Δ*sgl1.* Total lung tissue homogenates (n=3 mice/strain/timepoint) were analyzed using a Multiplex Elisa Array on days 2, 3, 4, 5, 6. and 14 post infection for various cytokines **(A–E, J, K)** and chemokines **(F–I)**. Significance was determined by a two-way ANOVA using Šídák’s multiple comparisons test for *P* value adjustment. Graphed data represents the mean +/- SD, and significance is denoted as *P < 0.05; **P < 0.01; ***P < 0.005; ****P < 0.001.

Since SGs were present in the BAL fluid post inoculation with *C. neoformans* Δ*sgl1*, we asked whether the level of SGs was associated with a protective cytokine milieu in the lungs. Indeed, we found that mice inoculated with *C. neoformans* Δ*sgl1* exhibited an early and significant increase in pro-inflammatory type 1/17 cytokines (**Figures 2A–E**) and chemokines (**Figures 2F–I**) in the lungs compared to *C. neoformans* WT-infected mice. Mice inoculated with *C. neoformans* Δ*sgl1* also exhibited a delayed and attenuated expression of type 2 cytokines compared to WT-infected mice who exhibited a strong type 2 immune cytokine signature (**Figures 2J, K**). Finally, mice inoculated with *C. neoformans* Δ*sgl1* displayed a resolution of the pro-inflammatory signals to baseline levels by day 14 post inoculation compared to WT-infected mice (**Figure 2**). Additionally, histopathology of mice lungs inoculated with *C. neoformans* Δ*sgl1* displayed high levels of inflammation at day 6 with visible yeast cells but returned to a less inflamed state by day 14 with no yeast visible (**Supplementary Figure S1**). This contrasts with the WT-infected mice that showed increased lung inflammation from day 6 to 14 with visible yeast still in the lung parenchyma. Overall, these data suggest that *C. neoformans* Δ*sgl1* induces an early and robust pro-inflammatory host immune response in the lungs that decreases as the mutant strain is cleared.

### *C. neoformans Δsgl1* Immunization Induces Robust Recruitment of Innate Effector Leukocytes to the Lungs

**Figure 3:**
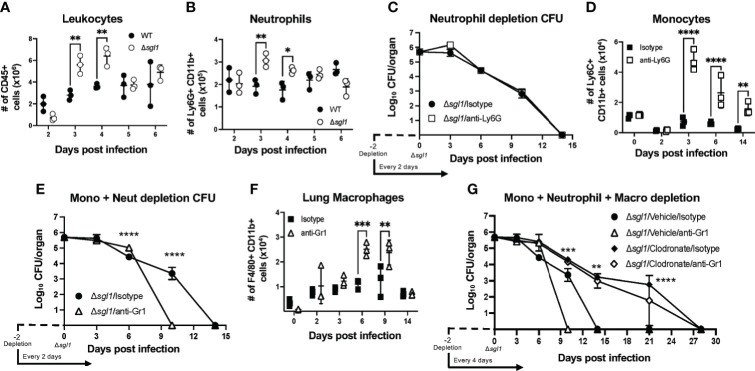
*C. neoformans (Cn)* Δ*sgl1* induces an increased recruitment of effector leukocyte and is cleared from the lung during combined immunosuppression. **(A, B)** Mice were intranasally injected with either 5x10^5^ *Cn* WT (black dots) or *Cn* Δ*sgl1* (white dots) and CD45^+^ leukocytes **(A)** or neutrophils **(B)** were quantified at various timepoints during the infection. **(C)** Lung fungal burden during inoculation with *Cn* Δ*sgl1* treated with either anti-Ly6G (neutrophil depleted) or isotype (control) antibody. **(D)** Monocytes were quantified in conditions of immunocompetentency (isotype antibody) or immunodeficiency (depletion antibody) after inoculation with *Cn* Δ*sgl1* **(E)** Lung fungal burden during infection with *Cn* Δ*sgl1* treated with either anti-Gr1 (monocyte and neutrophil depleted) or isotype (control) antibody. **(F)** Alveolar macrophages were quantified in conditions of immunocompetentency or immunodeficiency after inoculation with *Cn* Δ*sgl1.* **(G)** Lung fungal burden during infection with *Cn* Δ*sgl1* treated with either clodronate liposomes (alveolar macrophage depleted), clodronate and anti-Gr1 (alveolar macrophage, monocytes and neutrophil depletion), or isotype antibody/vehicle liposomes (control). All details of dosing frequency, antibody clone, or clodronate liposome configuration can be found in the material and methods section. Data shown represent the mean ± SD using 3 mice/group/timepoint. Significance was detemnined by a two-way ANOVA using Šídák’s multiple comparisons test for *P* value adjustment. For **(G)**, significance shown defines the comparison between *Δsgl1*/Vehicle/Isotype and Δ*sgl1*/Clodronate/Isotype. Significance is denoted as *P < 0.05; **P < 0.01; ***P < 0.005; ****P < 0.001.

Given that SGs were found in the airways alongside a strong inflammatory cytokine profile in the lungs post immunization with *C. neoformans* Δ*sgl1*, we next examined the recruitment of immune cells to the lungs during these early timepoints in response to the mutant. Mice were intranasally inoculated with either *C. neoformans* Δ*sgl1* or with the WT strain, and immune cell populations were quantified *via* flow cytometry (**Supplementary Figure S2**) at set timepoints post inoculation. We found that there was a significant increase in the total number of CD45^+^ leukocytes in the lungs on days 3 and 4 post inoculation with *C. neoformans* Δ*sgl1* compared to WT-infected mice (**Figure 3A**). Of these CD45^+^ leukocytes, we found that neutrophils were the major population recruited to the lungs (**Figure 3B**). These data suggest that *C. neoformans* Δ*sgl1* induces greater recruitment of innate effector cells to the lungs post immunization to promote pulmonary control and clearance.

### *C. neoformans Δsgl1* Is Cleared From the Lungs During Combined Innate Leukocyte Depletion

Because neutrophils were found to be the most abundant immune cell recruited to the lungs after *C. neoformans* Δ*sgl1* inoculation, we assessed host clearance of the mutant when neutrophils were depleted using the anti-Ly6G antibody (**Supplementary Figure S3A**). Neutrophil-depleted mice showed no difference in the rate of pulmonary fungal clearance compared to isotype-treated mice (**Figure 3C**), suggesting that neutrophils are not required for host clearance of *C. neoformans* Δ*sgl1*.

Flow cytometry of the lungs during neutrophil depletion showed that monocytes were significantly upregulated post *C. neoformans* Δ*sgl1* inoculation (**Figure 3D**). When we depleted both monocytes and neutrophils using the anti-Gr1 antibody (**Supplementary Figure S3B**), mice surprisingly cleared the mutant from the lungs at a faster rate than isotype-treated mice (**Figure 3E**), suggesting that Gr1^+^ cells are not required for host clearance of *C. neoformans* Δ*sgl1*.

Continuing with this same approach, flow cytometry analysis of Gr1-depleted mice showed that recruited macrophages (F4/80^+^ Ly6C^-^ SiglecF^-^ CD11b^+^) were significantly increased post *C. neoformans* Δ*sgl1* inoculation (**Figure 3F**). We utilized clodronate-loaded liposomes to deplete lung macrophages (**Supplementary Figure S3C**). Remarkably, mice treated with clodronate liposomes, with or without anti-Gr1 antibody depletion, were still able to clear *C. neoformans* Δ*sgl1*, although it took 28 days compared to the 14 days required by the control mice (**Figure 3G**). Nonetheless, even during the combined depletion of alveolar macrophages, neutrophils, and monocytes, mice were still able to eliminate *C. neoformans* Δ*sgl1* from the lungs. These data suggest that lung macrophages may promote early host recognition and control but are not required for sterilizing immunity to *C. neoformans* Δ*sgl1*.

### Host Clearance of *C. neoformans Δsgl1* Is Lost When CD4^+^ T Cells Are Depleted

**Figure 4:**
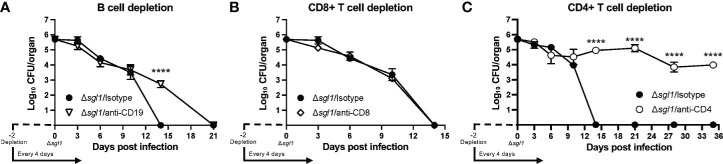
CD4^+^ T cells are necessary for clearance of *C. neoforrnans (Cn)* Δ*sgl1*. CBA/J mice were depleted of adaptive lymphocytes [**(A)**, B cells; **(B)**, CD8^+^ T cells; **(C)**, CD4^+^ T cells] and lung fungal burden analysis was determined for each condition. Significance was detemnined using a two-way ANOVA using Šídák’s multiple comparisons test for* P* value adjustment. All details of dosing frequency, antibody clone, or clodronate liposome configuration can be found in the material and methods section. Graphed data represents the mean*+I*-SD, and significance is denoted as ****P < 0.001.

Since even the combined loss of early innate effector cells was not sufficient to prevent host clearance of the mutant, we examined the role of B and T lymphocytes on the clearance of *C. neoformans* Δ*sgl1* using depleting antibodies (**Supplementary Figures S3D–F**). We found that mice depleted of B cells (**Figure 4A**) or CD8^+^ T cells (**Figure 4B**) were still able to fully clear the mutant. However, mice lacking CD4^+^ T cells were unable to clear *C. neoformans* Δ*sgl1* from the lungs by day 35 post inoculation (**Figure 4C**). Mutant fungal cells persisted in the lungs of CD4-depleted mice past the 30-day immunization period although there was a leveling out of the CFU with no increase in the pulmonary burden after day 14. Interestingly, these mice also displayed no physical symptoms of morbidity or extrapulmonary dissemination of the mutant to the brain, spleen, kidney, or liver over this timeline. These data ultimately suggest that depletion of CD4^+^ T cells resulted in the persistence of *C. neoformans* Δ*sgl1* from the lungs past the 30-day immunization period.

### Loss Of Both CD4^+^ And CD8^+^ T Cells Results in a Complete Loss of Protection to WT Challenge After *C. neoformans Δsgl1* Immunization

**Figure 5:**
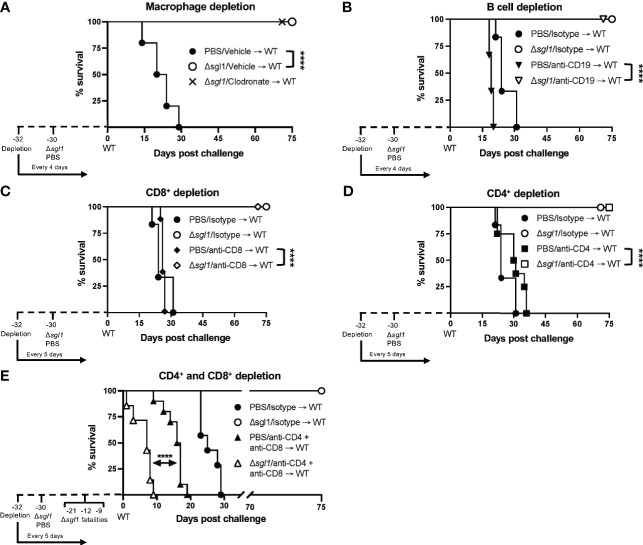
Vaccination with *C. neoformans (Cn)* Δ*sgl1* requires either CD4^+^ or CD8^+^ T cells for complete protection against lethal WT challenge. CBA/J mice (n=10 mice/group) were depleted of specific cell types 48 hours prior to intranasal immunization with 5x10^5^ Cn Δ*sgl1* or PBS for unvaccinated controls, and depletions continued for the entirety of the experiment at set intervals. Mice were challenged with 5x10^5^ WT strain 30 days post immunization and assessed for survival until day 75 post challenge. Mice administered PBS (unvaccinated controls) are denoted in black and *Cn* Δ*sgl1* denoted in white. Significance was determnined by the Mantel-Cox log-rank test. Significance is denoted as ****P < 0.001.

We have previously shown that *C. neoformans* Δ*sgl1* confers complete protection against the WT infection in immunocompetent or CD4^+^ T cell deficient hosts (Rella et al., 2015). Thus, we asked if *C. neoformans* Δ*sgl1*-vaccinated mice are still protected from lethal WT challenge under other models of immunodeficiency. To answer this, we utilized an antibody depletion approach in which we depleted macrophages, B cells, CD8^+^ T cells, CD4^+^ T cells, or CD4^+^ and CD8^+^ T cells before immunization with *C. neoformans* Δ*sgl1* and continued immune cell depletion throughout the end of the experiment. Remarkably, *C. neoformans* Δ*sgl1*-vaccinated mice depleted of alveolar macrophages (**Figure 5A**), B cells (**Figure 5B**), CD8^+^ T cells (**Figure 5C**), or CD4^+^ T cells (**Figure 5D**) maintained complete protection against WT challenge with 100% survival. Endpoint tissue burden culture (TBC) analysis revealed absolutely no extrapulmonary dissemination of the yeast in vaccinated mice with no fungal cells found in the brain, spleen, liver, or kidneys (**Supplementary Figure S4**). *C. neoformans* WT cells were still found in the lungs of all mice (isotype or antibody depleted), yet no *C. neoformans* Δ*sgl1* cells were recovered from any organ, even from the lungs of CD4-deficient mice where the mutant persisted past the 30-day immunization period indicating that the mutant was cleared post WT challenge. Moreover, histopathology showed the host contained the persistent WT cells in localized regions in the lungs (**Supplementary Figure S5**). Lungs from *C. neoformans* Δ*sgl1-*vaccinated mice were taken at days 45, 75, and 105 post WT challenge, and there was an obvious decrease in lung inflammation with progressive granulomatous regions in areas that still contained yeast cells. These data strongly indicate that *C. neoformans* Δ*sgl1-*vaccinated mice control the persistent WT cells without any concern for dissemination. Ultimately, our data suggest that *C. neoformans* Δ*sgl1* immunization may be a viable vaccine option under immunodeficient conditions when one cell type is lost, and that protection is not dependent on prior clearance of the mutant or the WT strain post challenge.

However, depletion of both CD4^+^ and CD8^+^ T cells resulted in a complete loss of protection (**Figure 5E**). Moreover, three mice succumbed to infection during the *C. neoformans* Δ*sgl1* immunization phase (days -21, -12, -9 post WT challenge), and TBC revealed that these mice had *C. neoformans* Δ*sgl1* in all organs (data not shown). The remaining vaccinated mice succumbed to infection within 10 days post WT challenge, which was significantly quicker than the unvaccinated control group (**Figure 5E**). Due to the lack of control and early death, we further confirmed our depletion in TCR β KO mice inoculated with *C. neoformans Δsgl1*, which are genetically devoid of both CD4^+^ and CD8^+^ T cells. Indeed, these mice all succumbed to the primary inoculation with *C. neoformans* Δ*sgl1* by day 19, yet all C57 mice cleared the infection by day 14 with no dissemination from the lungs (**Supplementary Figure S6**). Overall, these data suggest that *C. neoformans* Δ*sgl1*-mediated host protection to the WT strain requires either CD4^+^ or CD8^+^ T cells.

## Discussion

In our current study, we have shown that *C. neoformans* Δ*sgl1* elicits a robust, pro-inflammatory host response in the lungs that stimulates the clearance of the mutant ultimately leading to subsequent protection from a lethal WT challenge. We were able to measure significantly increased levels of SGs during *in vivo* inoculation with the mutant, providing evidence that SGs are present outside the yeast and may be the driving factor of host immunomodulation. This pro-inflammatory host response was sufficient to clear the mutant from the lungs during combined immunosuppressive conditions as well as protect the host from a lethal WT challenge in the presence of either CD4^+^ or CD8^+^ T cells.

Notably, we determined that SGs were found *in vivo* during inoculation with *C. neoformans* Δ*sgl1* but not the WT strain (**Figure 1C**). These SGs were believed to cause the significantly increased levels of Th1 cytokines, including IL-1α, TNFα, IL-17, and IFNγ, and decreased levels of Th2 cytokines, including IL-4 and IL-5 (**Figure 2**) since they occurred over the same timeline as the SGs were measurable *in vivo* (**Figure 1C**). It is widely accepted that host protection against *C. neoformans* requires a pro-inflammatory type 1 immune response mediated by IFNγ (Mourad and Perfect, 2018; Li et al., 2019; Linyu et al., 2020; Hester et al., 2020; Normile et al., 2020a; Akhtar et al., 2020; Montoya et al., 2021). Interestingly, SGs have been shown to induce Th1 T cell polarization (Bouic et al., 1996; Lee and Han, 2006; Lee et al., 2007), but these studies have used plant-based SGs, not fungal-derived SGs. To our knowledge, this is the first report of fungal-derived SGs being used as an immunoadjuvant inducing a robust type 1 cytokine milieu in the lungs for protective host immunity.

Our SG-accumulating mutant also induced increased effector leukocyte and adaptive lymphocyte recruitment to the lungs compared to WT infected mice (**Figure 3A**). This, too, temporally correlated with the levels of SGs measured *in vivo* as well as the cytokine profile in the lungs since we observed significantly increased levels of chemotactic chemokines such as G-CSF, KC (CXCL1), MIP-1b (CCL4), and RANTES (CCL5) (**Figure 2**). Our increased neutrophil, monocyte, and macrophage response post inoculation with *C. neoformans* Δ*sgl1* correlated with the same timeline as these leukocyte chemotactic chemokines. Moreover, the significantly increased levels of RANTES (CCL5) from days 4 to 6 post inoculation with *C. neoformans* Δ*sgl1* correlated with strong recruitment of lymphocytes to the lungs yielding a robust adaptive immune response, which is a known effect of SGs seen previously in the literature using other forms of this glycolipid (Lee and Han, 2006; Lee et al., 2007).

Interestingly, these chemotactic factors also decrease to baseline levels by day 14 post inoculation with *C. neoformans* Δ*sgl1* displaying a resolution of infection correlating with the same time frame for clearance of the mutant strain (and lack of measurable SGs), suggesting a delayed-type hypersensitivity host immune response. This was opposite to what was observed for the WT strain, where the chemotactic factors and pro-inflammatory cytokines were delayed and increased later post infection around day 14 resulting in host pathology. Lung histology confirmed these data showing the lungs of *C. neoformans* Δ*sgl1* inoculated mice displayed a decrease in inflammation from day 6 to 14 during the time the mutant was cleared, cytokine levels decreased, and leukocyte recruitment halted (**Supplementary Figure S1**). Overall, we concluded the increased levels of SGs derived from our mutant sparked these positive host conditions and downstream protective nature observed during the WT challenge.

One interesting caveat that was recently published by our group has shown that *C. neoformans* Δ*sgl1* does have a growth defect in low oxygen and glucose conditions (de Sa et al., 2021). We cannot overlook the fact that the lung interstitium is anoxic compared to the alveolar spaces, and the hypoxia induced death of the mutant may be the causative reasoning for the proinflammatory environment with increased leukocyte migration. However, we do not believe this to be the case as the mutant strain would be unable to persist in the lungs to the day 35 timepoint in the CD4-deficient conditions (**Figure 4C**) and past the WT challenge in CD4- and CD8-dual deficiency (**Figure 5E**). We do believe the fungal SGs in combination to the yeast cells induce the augmented host responses observed that result in the mutant clearance and host vaccination to the WT strain.

We utilized an *in vivo* antibody depletion strategy to assess the necessity of specific cell types for host clearance of *C. neoformans* Δ*sgl1*. Interestingly, we found that only CD4^+^ T cells were the only cell type examined that delayed host clearance of the mutant during the 30-day immunization period (**Figures 3**, **4**). Solo or combined innate effector leukocyte depletion (neutrophils, monocytes, and/or macrophages), as well as solo adaptive lymphocyte depletion (B cells and CD8^+^ T cells), resulted in clearance of the mutant within the 30-day immunization period, although at various times compared to the isotype-treated mice. Although the mice were unable to clear the infection in the absence of CD4^+^ T cells, no morbidity or extrapulmonary dissemination was observed and the lung CFU remained relatively stable from day 10 to day 35 (**Figure 4C**) strongly suggesting that mice can control the mutant without displaying sterilizing immunity.

The keystone factor of our mutant strain is the lasting vaccine protection for the host against lethal WT infection shown previously (Rella et al., 2015). We have added to the robustness of this protection beyond just CD4^+^ T cell depletion. Mice immunized with *C. neoformans* Δ*sgl1* depleted of either alveolar macrophages, B cells, or CD8^+^ T cells have all shown complete host protection (**Figure 5**) without any extrapulmonary dissemination (**Supplementary Figure S4**). We have also uncovered an interesting facet of our mutant in that complete host protection was not requisite of prior mutant clearance shown by the protection in CD4-depleted mice. Although we had previously reported that *C. neoformans* Δ*sgl1* conferred complete protection in CD4-deficient mice, we have now uncovered these mice do not clear the mutant prior to WT infection. The mounted host immune response against the WT challenge was sufficient to clear *C. neoformans* Δ*sgl1* from the lungs. This was the opposite for the WT cells that remain in the lungs for at least 75 days post challenge in all mice tested, whether immunocompetent or immunocompromised (**Supplementary Figure S4**). Nonetheless, these mice show no signs of morbidity or extrapulmonary dissemination, so we attest these mice are controlling replication and dissemination of the yeast. This statement of controlling the WT yeast post challenge was supported with histological data (**Supplementary Figure S5**). The degree of lung tissue inflammation decreased from day 45 post challenge to 105 post challenge even though no significant changes in lung CFU were observed over this timeline. The WT yeast were shown to be contained within granulomatous regions in the lung, but not within healthy tissue, strongly suggesting a controlled means of host containment. Exploring the immunological niche of these granulomatous regions and clearance of the WT strain from the lungs will be an interesting topic for future experimentation and currently underway in our lab.

Our data strongly suggest that either CD4^+^ or CD8^+^ T cells are required for control of *C. neoformans* Δ*sgl1* (**Figure 5E** and **Supplementary Figure S6**) and the conferred complete host protection post vaccination (**Figure 5E**). When either of these subsets are depleted alone, full control of the mutant strain (**Figure 4**) and the WT strain post challenge (**Figure 5**) are observed with no visible pathology or extrapulmonary dissemination (**Supplementary Figure S4**). The immunological roles of CD4^+^ and CD8^+^ T cells are not normally redundant but have been shown to be complementary during the absence of the other subset in other work (Lindell et al., 2005; Nanjappa et al., 2012a; Nanjappa et al., 2012b). We conclude here that the roles of CD4^+^ and CD8^+^ T cells are redundant and overlapping post vaccination with *C. neoformans* Δ*sgl1* while the other subset is missing. However, we hypothesize that CD4^+^ T cells are more robust in their protective nature since we observed that clearance of *C. neoformans* Δ*sgl1* was not accomplished in CD4-deficient hosts (**Figure 4C**), and the endpoint CFU after 75 days post challenge in CD4-deficient hosts was significantly greater than that of the isotype-treated mice (**Supplementary Figure S4C**). The immunological roles of these cells in immunocompetent and immunosuppressed hosts are currently being explored in our lab.

In conclusion, *C. neoformans* Δ*sgl1* demonstrates a viable option as a future therapeutic for protection against cryptococcosis, especially due to the fact of being tested under conditions of severe immunosuppression. This is principally due to three main factors: i) the early beneficial properties initiated in the host seen by the lung cytokine profile and early effector cytokine recruitment (**Figures 2** and **3A–B**); ii) the clearance and lack of extrapulmonary dissemination of the mutant during solo or combined immunosuppression (**Figures 3C–G** and **4**); and iii) the host protection against lethal WT challenge during several solo models of immunosuppression without the necessity for initial host clearance of *C. neoformans* Δ*sgl1* (**Figure 5** and **Supplementary Figure S4**). Additionally, the notable effect observed by fungal-derived SGs on the host immune response demonstrated a novel immunoadjuvant effect not previously shown before. However, it should be noted that this immunoadjuvant requires T cell priming for control and protection as highlighted through our dual CD4^+^ and CD8^+^ T depletion and TCR β KO mice (**Figure 5E** and **Supplementary Figure S6**), so pan T cell immunosuppression would be a limiting factor of our mutant from a clinical viewpoint. Nonetheless, because we show that either CD4^+^ or CD8^+^ T cells are required, *C. neoformans* Δ*sgl1* still remains a viable option for the major risk factors during the opportunistic infection with *C. neoformans* since CD8^+^ T cells remain accessible during CD4-lymphopenia (Collins et al., 2020; Akhtar et al., 2020; Linyu et al., 2020). We are also currently investigating subunit-based vaccination strategies that have the potential to surpass this limitation. Overall, our data strongly suggest that fungal-derived SGs promote beneficial host immunity during the primary infection when SGs are present and leave a lasting immune signature on protection against a normally lethal WT challenge when SGs are absent. Future experimentation on direct host recognition of these fungal SGs and exploring the roles of non-canonical T cells in the protection by our mutant will be exciting points to explore.

## Data Availability Statement

The datasets presented in this study can be found in online repositories. The names of the repository/repositories and accession number(s) can be found in the article/**Supplementary Material**.

## Ethics Statement

The animal study was reviewed and approved by IACUC Stony Brook University.

## Author Contributions

TGN, AR, and MDP took part in the conceptualization of this study. TGN performed animal infections, depletions, survival studies, CFU quantification, and flow cytometry analysis. AR performed the cytokine determination and SG quantification experiments. All authors contributed to the article and approved the submitted version.

## Funding

This work was supported by NIH grants AI136934, AI116420, and AI125770, and by the Merit Review Grant I01BX002924 from the Veterans Affairs Program to MDP. MDP is a Burroughs Welcome Investigator in the Pathogenesis of Infectious Diseases and a Research Career Scientist at the Veterans Administration Medical Center in Northport, NY.

## Conflict of Interest

MDP is a Co-Founder and Chief Scientific Officer (CSO) of MicroRid Technologies Inc.

The remaining authors declare that the research was conducted in the absence of any commercial or financial relationships that could be construed as a potential conflict of interest.

## Publisher’s Note

All claims expressed in this article are solely those of the authors and do not necessarily represent those of their affiliated organizations, or those of the publisher, the editors and the reviewers. Any product that may be evaluated in this article, or claim that may be made by its manufacturer, is not guaranteed or endorsed by the publisher.
