# Supplementary material for: Cryptococcus neoformans Δsgl1 Vaccination Requires Either CD4+ or CD8+ T Cells for Complete Host Protection
Source: Front Cell Infect Microbiol. 2021 Sep 8;11:739027. doi: 10.3389/fcimb.2021.739027 (PMC8455912; doi:10.3389/fcimb.2021.739027)
Supplement: Supplementary file 1 [file DataSheet_1.pdf]

Supplementary Material

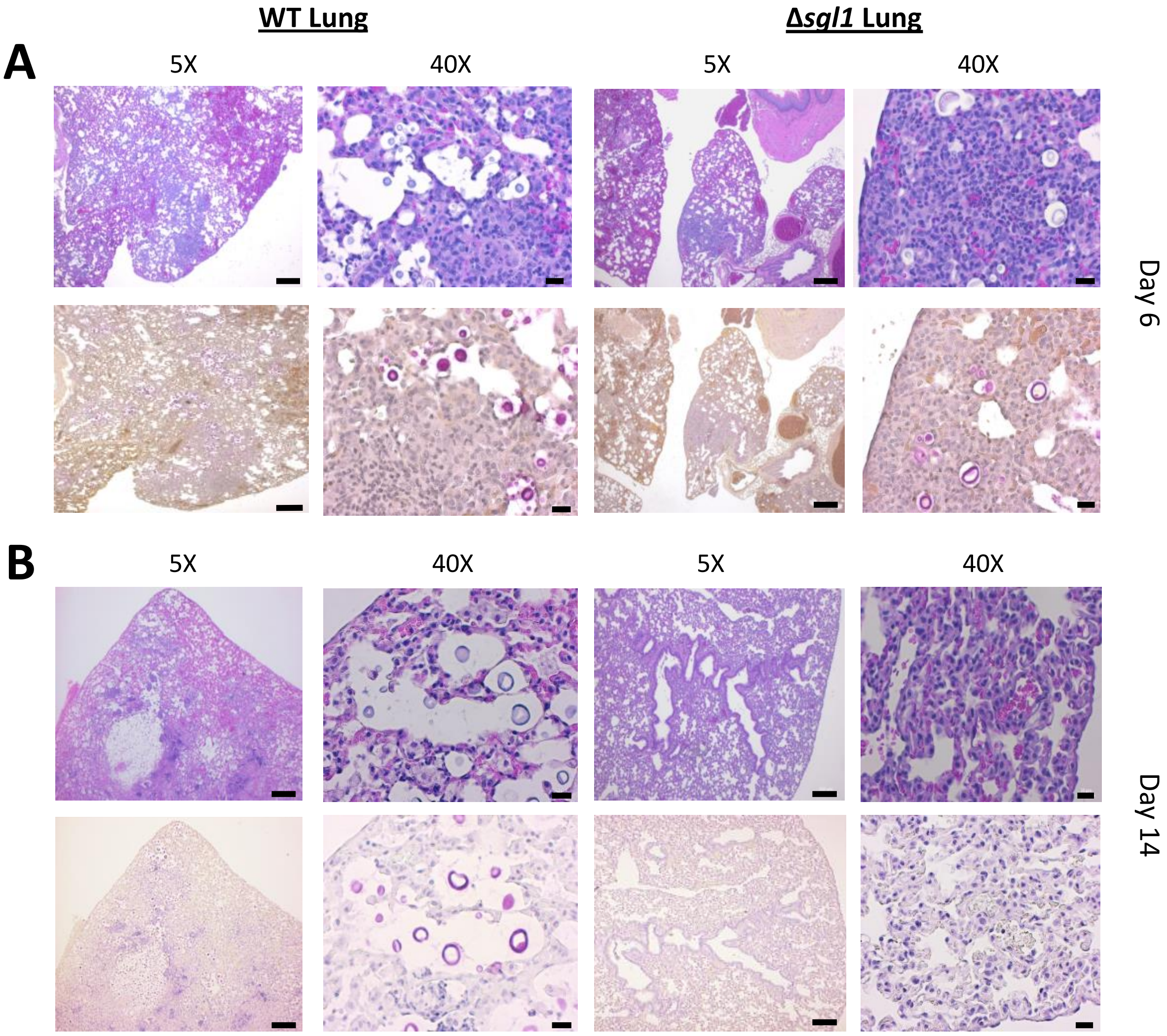

Supplementary Figure S1. Lung sections of mice infected with *C. neoformans* WT and Δ*sgl1* obtained at 6- and 14-days post infection. The presence of the yeast is found in the lungs of mice infected with WT at 6- and 14-days post infection, whereas the Δ*sgl1* mutant is present in the lungs at day 6 but cleared by day 14 with decreasing inflammation. The lung sections were stained with H&E (upper panels), and mucicarmine (lower panels). For the top row, the scale bar = 200µm. For the bottom row, the black scale bar = 50µm.

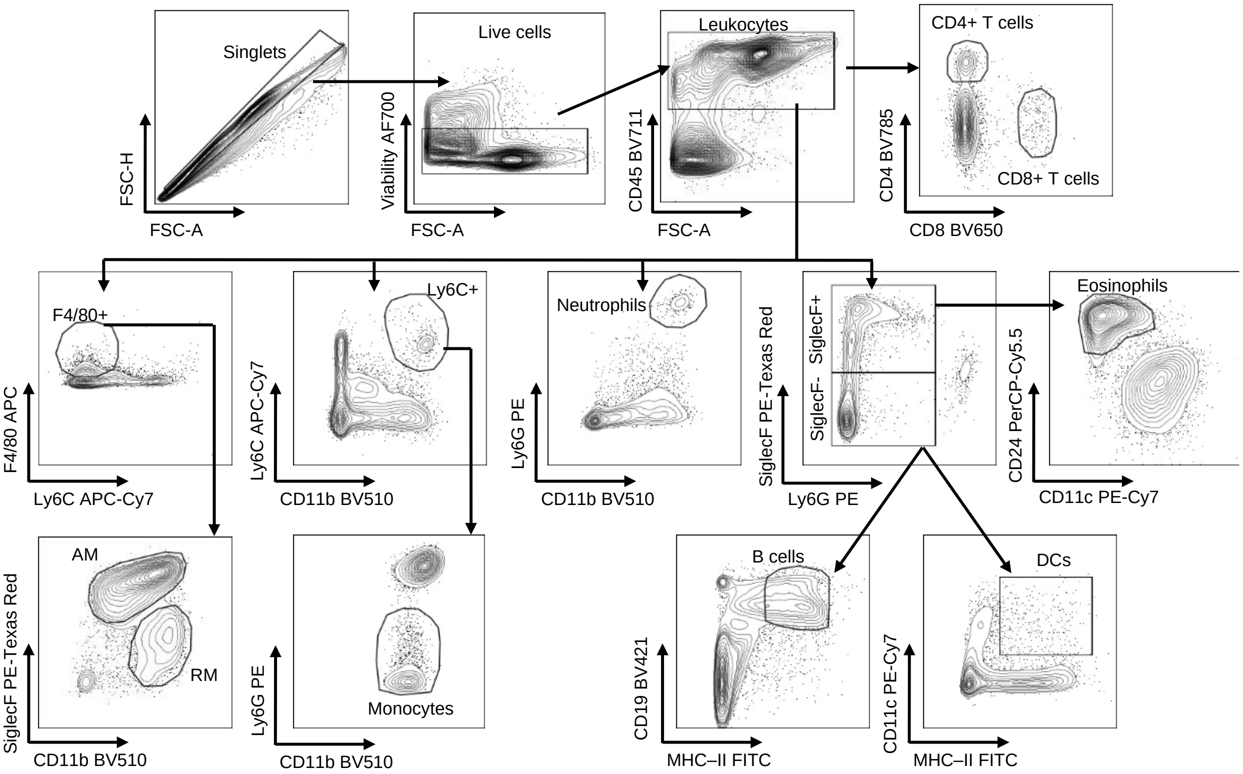

Supplementary Figure S2. Representative gating strategy for leukocyte populations in mouse lungs. From live, CD45^{+} leukocytes, the following cell populations were quantified: CD4^{+} T cells (CD4^{+} CD8^{-}), CD8^{+} T cells (CD8^{+} CD4^{-}), alveolar macrophages (AM) (F4/80^{+} Ly6C^{-} Siglec F^{+} CD11b$^{+/-}$), recruited macrophages (RM) (F4/80^{+} Ly6C^{-} Siglec F^{-} CD11b^{+}), monocytes (Ly6C^{+} CD11b^{+} Ly6G^{-}), neutrophils (Ly6G^{+} CD11b^{+}), eosinophils (Siglec F^{+} CD24^{+} CD11c^{-}), B cells (Siglec F^{-} CD19^{+} MHC-II^{+}), and dendritic cells (DCs) (Siglec F^{-} CD11c^{+} MHC-II^{+}).

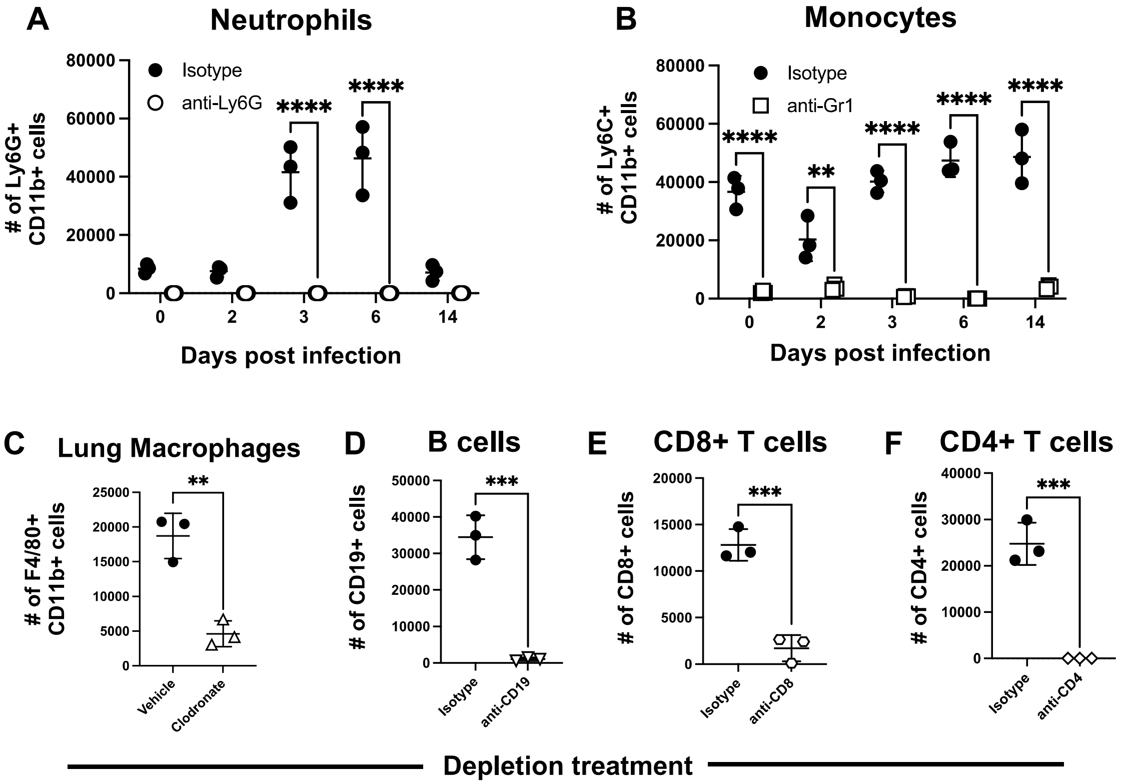

Supplementary Figure S3. *In vivo* antibody and liposome depletion confirmed by flow cytometry. Neutrophils were depleted with anti-Ly6G administered IP (**A**), monocytes and neutrophils with anti-Gr-1 administered IP (**B**), AM with clodronate-loaded liposomes administered IN (**C**), B cells with anti-CD19 administered IP (**D**), CD8 T cells with anti-CD8 IP (**E**), and CD4 T cells with anti-CD4 administered IP (**F**). Proper IgG isotype antibodies or empty liposomes were used for each experiment. Details of dosing frequency, antibody clone, or clodronate liposome configuration can be found in the material and methods section. Data are representative of mean ± SD using 3-6 mice/group/timepoint and significance was determined via an unpaired t-test. (*, $P < 0.05$; **, $P < 0.01$; ***, $P < 0.005$; ****, $P < 0.001$).

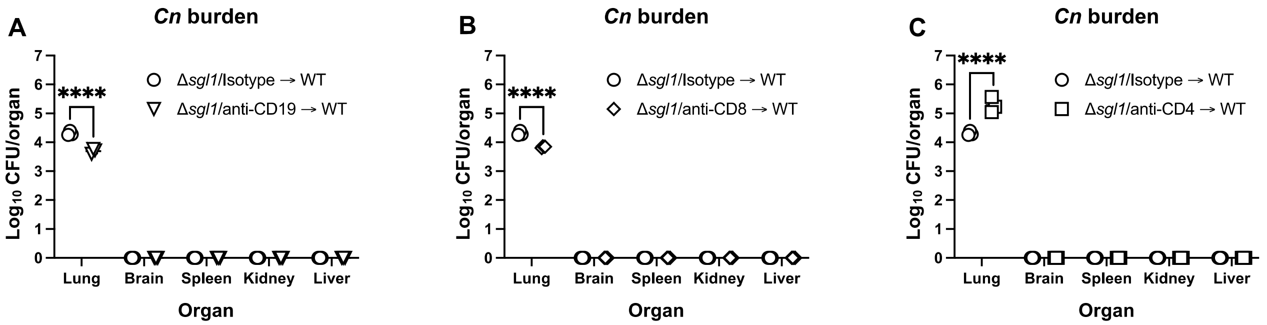

Supplementary Figure S4. Endpoint fungal burden in the lungs, brain, spleen, kidney, and liver of *C. neoformans (Cn)* Δ*sgl1*-vaccinated mice surviving the WT infection 75 days post challenge. Data shown represent the mean ± SD using 3-4 mice/group. Significance was determined by a two-way ANOVA using Šídák's multiple comparisons test for *P* value adjustment, and significance is denoted as *, $P < 0.05$; **, $P < 0.01$; ***, $P < 0.005$; ****, $P < 0.001$.

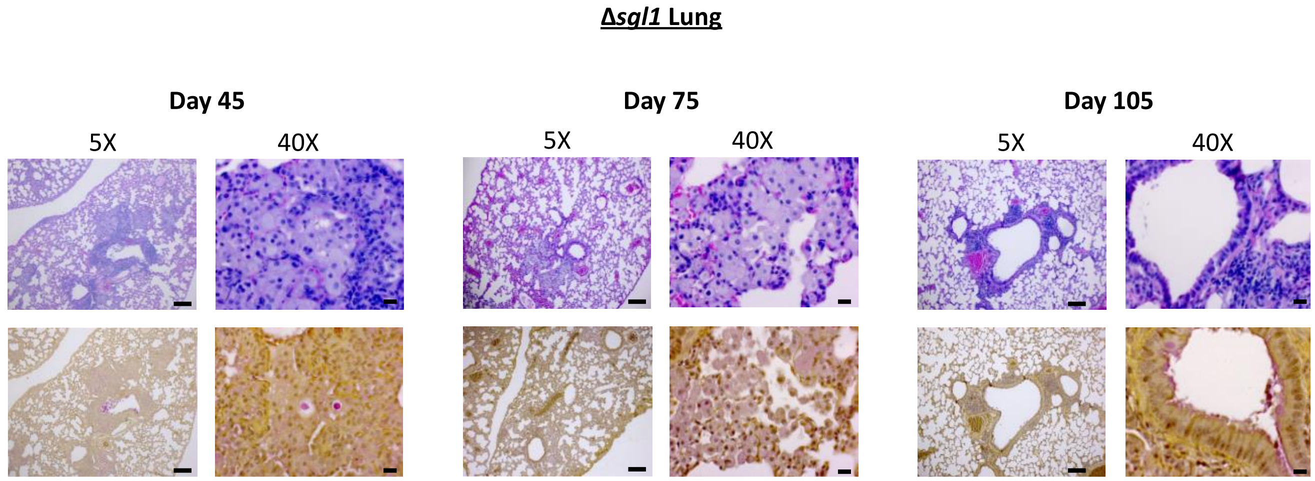

Supplementary Figure S5. Mice immunized with *C. neoformans Δsgl1* do not clear the WT yeast post challenge but display a progressive granulomatous response for containment. Mice were immunized with *C. neoformans* Δ*sgl1* 30 days prior to challenge with *C. neoformans* WT. Three lungs at each timepoint were isolated for histology at 45-, 75-, and 105-days post WT challenge. The lung sections were stained with H&E (upper panels), and mucicarmine (lower panels). *C. neoformans* cells stain bright magenta whereas cellular mucin stains light pink in mucicarmine. For the top row, the scale bar = 200µm. For the bottom row, the black scale bar = 50µm.

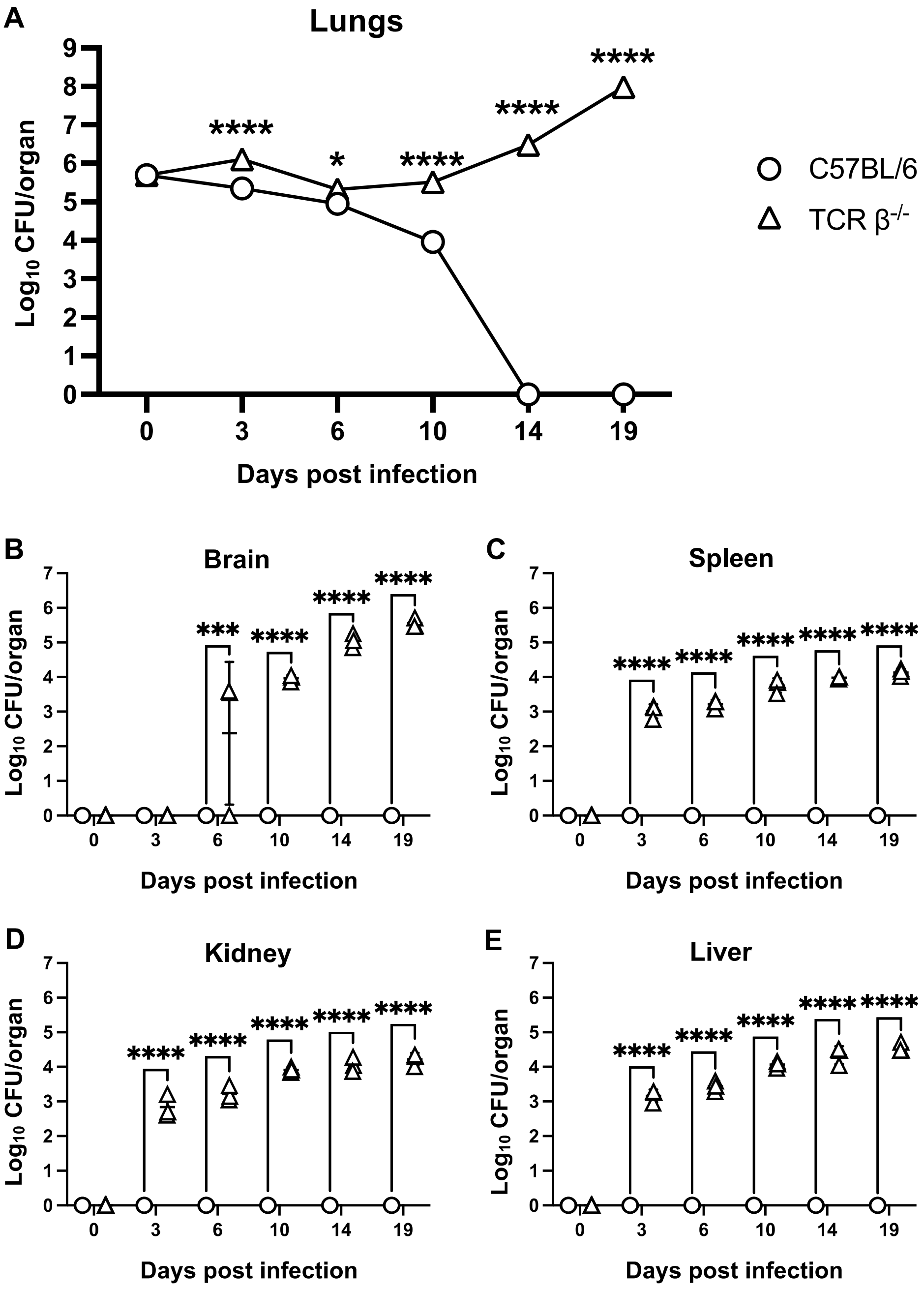

Supplementary Figure S6. TCR $\beta^{-/-}$ mice fail to control IN inoculation with *C. neoformans* Δ*sgl1*. C57BL/6 or TCR $\beta^{-/-}$ were inoculated with 5x105 CFU *C. neoformans* Δ*sgl1* and organ burden was assessed at various timepoints in the lungs (**A**), brain (**B**), spleen (**C**), kidney (**D**), and liver (**E**). Data shown represent the mean ± SD using 3-4 mice/group. Significance was determined by a two-way ANOVA using Šídák's multiple comparisons test for *P* value adjustment, and significance is denoted as *, $P < 0.05$; **, $P < 0.01$; ***, $P < 0.005$; ****, $P < 0.001$.
